# Inductive prediction in biology: Are long‐term ecological and evolutionary processes predictable?

**DOI:** 10.15252/embr.202254846

**Published:** 2022-06-20

**Authors:** Valentí Rull

**Affiliations:** ^1^ Botanic Institute of Barcelona Barcelona Spain

## Abstract

Reliable predictions of long‐term ecological and evolutionary processes require more information than the periodicity of the astronomical forces that drive them.

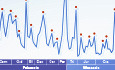

In an earlier essay (Rull, 2022a), I discussed the possibility of making inductive predictions in biology and that this approach is hampered by the lack of fundamental biological laws that can be described mathematically, as is usual in physics. The development of such laws, in turn, is thwarted by the nature of life and its evolution both of which are characterized by emergent properties such as complexity, contingency and unpredictability. Biological induction – and thereby knowledge – is largely based on generalization after hypothesis testing, an approach that has also been called accommodation. I also argued that there is no reason to underrate accommodating procedures with respect to inductive prediction, a usual practice in theoretical biology.

However, even in the absence of fundamental laws, it might be possible to develop a logical, usually mathematical, description for biological processes that could serve as a basis for physics‐like inductive predictions. In this essay, I discuss this potential for predicting some evolutionary and long‐term ecological processes. The emphasis is on periodic long‐term processes, as these have the highest predictive potential owing to their potentially quantifiable recurrence. It is important to differentiate this approach from evidence‐based predictive modelling, where the model is the hypotheses and is progressively refined/reframed to accommodate empirical observations. In contrast, inductive prediction relies on immutable underlying laws and uses these to predict empirical observations that would prove the validity of such rules; for instance by finding evidence for predicted evolutionary processes in the fossil record (Rull, 2022a).

The fossil record already provides us with evidence for biotic responses to repeated environmental events on a diurnal to million‐year scales. Four main bands of recurrent environmental variability have so far been defined: calendar (< 1 year), solar (1–10^4^ years), Milankovitch (10^4^–10^6^ years) and galactic (> 10^6^ years) (Rodríguez‐Tovar, 2014). This paper focuses on the galactic and Milankovitch bands and the long‐term ecological and evolutionary responses. Within these time domains, the best‐known recurrent phenomena are the Phanerozoic extinction events during the past ∼540 million years (or Myr) – the causes of which are still under debate – and the Pleistocene global biotic reorganizations linked to glacial–interglacial cycles during the past 2.6 Myr.

…even in the absence of fundamental laws, it might be possible to develop a logical, usually mathematical, description for biological processes that could serve as a basis for physics‐like inductive predictions.

## Phanerozoic cycles of extinction

The hypothesis by the American palaeontologists Raup & Sepkoski (1984) that Phanerozoic extinctions occur periodically and that they are linked to external events has been intensively debated. They identified 12 extinction pulses during the past 250 Myr with an average periodicity of 26 Myr and suggested that this periodic behaviour could be linked to solar or galactic cycles. The authors based their hypothesis on Fourier analysis of a time series of fossil records encompassing ∼3,500 families of marine animals – vertebrates, invertebrates and protozoans – compiled by one of the authors (Sepkoski) and the Harland and Odin geological timescales, which were the best developed at the time. The differences between the observed extinction peaks and those predicted by the 26‐Myr harmonic function ranged between 0 and 6 Ma for the Harland scale and 0.6 and 10.6 Myr for the Odin scale. The debate started when other scientists criticized Raup and Spekoski's analysis on the basis of taxonomic, chronological and statistical deficiencies. Both repeated their analysis over the following 4 years and confirmed the 26‐Myr periodicity, although they admitted that more data were needed to resolve the controversy.

**Figure 1:**
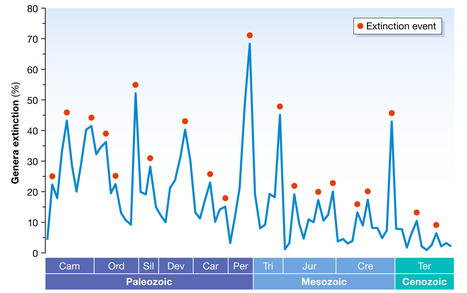
Extinction events from 540 Ma to present (red dots) The average duration of the extinction cycles – the interval between consecutive peaks – is ~ 27 Myr. Cam, Cambrian; Ord, Ordovician; Sil, Silurian; Dev, Devonian; Car, Carboniferous; Per, Permian; Tri, Triassic; Jur, Jurassic; Cre, Cretaceous; Ter, Tertiary. Based on Melott & Bambach (2014) and literature therein.

Further attempts using improved databases and updated timescales extended to the last 500 Myr gave similar periodicities ranging from 27 to 30 Myr (Melott & Bambach, 2014). This paper used an updated time‐series database (Fig 1) and a new paleobiology database (PBDB) and identified two types of periodicities in the recurrence of extinction: ∼27 Ma and ∼62 Myr (both with errors of ± 3 Myr), acting together in and out of phase during the past 470 Myr. Although the authors did not favour any particular cause – neither for the extinction events nor for their periodicity – others did associate periodic extinctions with astronomical causes, such as the passage of the Solar System through the galactic plane, which has a ∼63‐Myr periodicity (Gillman & Erenler, 2017). However, these conclusions were not universally accepted. For example, Erlykin *et al* (2017) used an updated time‐series database and Fourier analysis and concluded that no statistically significant periodicities exist in the extinction record for the past ∼500 Myr. These authors dismissed astronomical causes of extinction events and favoured other mechanisms, such as asteroid impacts, climatic changes and plate tectonic processes.

There is a tendency of linking long‐term periodic patterns to astronomical causes given the latter's periodic nature, but reliable evidence of this relationship remains elusive. Other proposed causes for extinctions, such as meteorite impacts, global biogeochemical shifts, volcanism, reversals in Earth's magnetic field or the formation of large igneous provinces (LIPs) caused by massive magma outbreaks, are more difficult to associate with periodic phenomena. However, some of these causes may also have a periodic component. For example, the formation of LIPs owing to the release of mantle plumes (flood basalts) into the oceans causes major biogeochemical disruptions by releasing large amounts of sulphates and carbon dioxide with significant environmental and biotic consequences at a global level. Using cross‐wavelet and other time‐series analyses, Prokoph *et al* (2013) found a periodicity of 62–65 Myr for this phenomenon during the past ∼500 Myr, which is consistent with the pattern of extinctions among marine organisms.

There is a tendency of linking long‐term periodic patterns to astronomical causes given the latter's periodic nature, but reliable evidence of this relationship remains elusive.

If we assume that extinction events are periodic, it should be possible to make two types of inductive predictions: the timing of the next extinction event and the timing of a hypothetical past event still unnoticed in the fossil record. For example, using the periodicity of 63 Myr for Solar System dynamics, Gillman & Erenler (2017) anticipated that the next extinction event should occur in 1–2 Myr. Curiously, this coincides with current extrapolations of extinction rates, although the causal mechanisms are radically different, being based on anthropogenic extinctions (Rull, 2022b). Regarding non‐astronomical causes, Prokoph *et al* (2013) used the periodicity of mantle plume releases (62–65 Myr) to predict a still unidentified extinction event 440–450 Myr ago.

Conversely, if periodicity is a methodological artifact and extinctions occur randomly (Erlykin *et al*, 2017), the potential for developing mathematical laws from the fossil record and inductive predictions would be significantly less. In that case, the best option would be falling back on accommodating approaches, that is, hypothesis testing including evidence‐based predictive modelling to make further generalization whenever possible. Some attempts have been made in this regard by modelling the biosphere as an ecosystem; these models seem to be able to reproduce extinction events from only internal functioning without the need for external forces (Likhoshvai & Khlebodarova, 2021).

## Pleistocene glacial–interglacial cycles

On another scale, it is clear that Earth has undergone glacial–interglacial cycles, at least during the Pleistocene, with drastic changes in average temperature. This is based on the analysis of the oxygen isotopic composition (δ^18^O) of planktonic foraminifer shells; as these are sensitive to temperature, it allows to reconstruct paleotemperature records from deep‐sea marine cores. In the 1970s, some palaeoceanographers noted that Pleistocene δ^18^O curves matched the so‐called Milankovitch cycles, which had been defined in the 1920s by the Serbian astronomer Milutin Milanković, who estimated long‐term variations in solar energy reaching the Earth depending on orbital parameters such as eccentricity (100‐kyr period), obliquity (41‐kyr period) and precession (23‐kyr period). It thus seemed logical to attribute the Pleistocene glacial–interglacial changes to these astronomical cycles (Imbrie & Imbrie, 1979).

**Figure 2:**
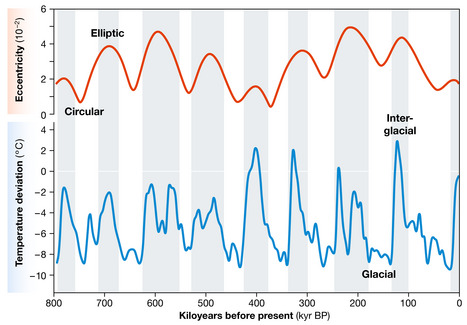
Changes in Earth's orbit eccentricity (red) compared with glacial–interglacial temperature shifts in the Antarctic (blue) for the past 800 kyr, during which a 100‐kyr periodicity has been dominated Earth's climate changes (Raymo, 1994) Lower eccentricity values (rounder orbit) imply lower solar radiation and are correlated with glaciations (white bands), whereas higher eccentricity values (more elliptical orbit) correspond to higher insolation values and are correlated with interglacial phases (grey bands). Redrawn and modified from Ellis & Palmer (2016).

Altogether, more than 100 climatic oscillations occurred during the Pleistocene, of which ∼40 reached the magnitude of glacial–interglacial cycles. These cycles show a clear periodicity of 41 kyr (obliquity) until 800 kyr before present and then change to a periodicity of 100 kyr (eccentricity) (Fig 2; Raymo, 1994). Although the Milankovitch cycles do match the pace of Pleistocene glacial cycles, the changes in the incoming solar energy seem to be insufficient to trigger glacial–interglacial cycles. The intensity of climatic changes is therefore explained by amplifying, nonlinear mechanisms, such as changes in albedo, thermohaline circulation or the concentration of atmospheric greenhouse gases (Ellis & Palmer, 2016).

Such a framework would mean a high predictive potential. For example, we could predict that the next glaciation will begin within a few thousand years given that the last glaciation occurred between about 110 and 20 kyr with a 100‐kyr periodicity. We can make a similar prediction – about 1,500 years from now – if we compare the Holocene, the interglacial period in which we live, with former interglacial phases of about the same duration (Tzedakis *et al*, 2012). However, the abovementioned amplifying mechanisms complicate things, especially as these are affected by human activities. The most important of these factors is the anthropogenic increase in carbon dioxide, which creates a greenhouse effect that could delay the next glaciation period by 40 kyr or more. Some predictions go even further and suggest that a global temperature increase of 2°C would be enough to provoke a cascade effect that triggers a long‐lasting state of ‘hothouse’ Earth without further glaciations (Steffen *et al*, 2018).

**Figure 3:**
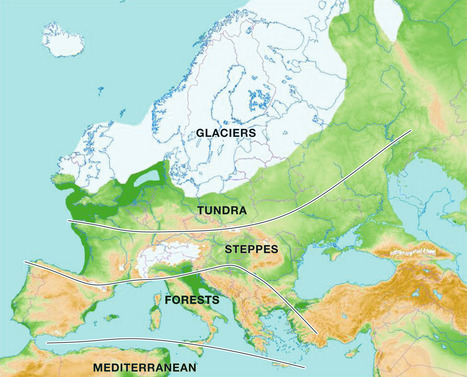
Southward migration and contraction of major European biomes during the last glaciation Most of the continent was covered by tundra and dry steppes, whereas the mixed and deciduous forests typical of today's central Europe were restricted to refugia of the southern peninsulas (Iberia, Italy, Greece). The Mediterranean biomes that today dominate these peninsulas were absent and restricted to northern Africa. Redrawn and modified from Rull (2020).

Glacial–interglacial cycles and the associated sea‐level shifts have affected the biosphere and ecological systems from the deep sea to the highest mountains. By way of example, the recurrent expansion (glacial) and contraction (interglacial) of polar ice masses, along with the repeated contraction and expansion of climatic zones and their corresponding biomes, have drastically influenced the terrestrial biota and its evolution (Fig 3). During glaciations, many species were restricted to residual areas, known as refugia, from which they expanded again during interglacials. Even in tropical zones, changes in precipitation patterns created refugia for many species that were driven by the glacial expansion of deserts. Moreover, significant glacial sea‐level changes of 100 m or more created new migration pathways or biogeographical barriers for terrestrial and marine organisms (Rull, 2020). The situation reversed during interglacials when geographical patterns were similar to those observed today.

… the response of forests with long‐lived trees to environmental changes can take centuries, which significantly inhibits any predictions of how the biota reacts to climate change.

However, translating the predictive potential of periodic climatic changes into biotic patterns is not straightforward owing to the large variability of how the biota responds to environmental changes. These responses are determined by idiosyncratic traits, such as life‐cycle duration, response lag, tolerance, phenotypic plasticity or the ability to migrate over longer distances. For example, foraminifers are single‐celled organisms with a short life cycle and their population immediately responds to temperature variations. This is why these organisms are excellent proxies for palaeotemperature reconstructions that provided empirical support to the orbital theory of Pleistocene climatic change. In contrast, the response of forests with long‐lived trees to environmental changes can take centuries, which significantly inhibits any predictions of how the biota reacts to climate change. Generally, organisms with a wider tolerance to temperature changes and/or high phenotypic plasticity are more able to acclimate and thereby survive, whereas stenothermic organisms are more sensitive. When climatic shifts overcome the acclimation capacity of organisms, species can still undergo adaptive evolution or migrate before going extinct. Yet, migration also differs among species owing to diverse propagation methods, migration velocities and changing spatial patterns, including the emplacement of refugia. All these factors affect the composition of ecosystems, to the effect that recurrent glacial–interglacial contractions and expansions have been accompanied with significant, but also stochastic and largely unpredictable modifications at the community level. The Pleistocene has also been an epoch of intense evolutionary change, notably at the species level, which further complicates the establishment of straightforward relationships between climate and the biota (Rull, 2020).

Thus, even in the case of periodic behaviour of Pleistocene climate cycles, the biological responses have not been homogeneous and straightforward, and a high degree of contingency and unpredictability remains. As a consequence, the glacial–interglacial shrinking–expansion cycles would be somewhat predictable at a biome level, but the taxonomic composition of biomes, their geographical distribution and the dynamic of their communities are largely unpredictable, even within a periodic palaeoclimatic framework. Practically, this means that it would be hard to make reliable predictions about either the reaction of the biota to future climate changes or about the timing and extent of past extinction events in response to climate change. The best solution is again the use of accommodating procedures, including the use of non‐inductive evidence‐based modelling of climate–biota relationships.

## Physical reductionism

At the temporal scales analysed here, periodicity, and hence predictability, is strongly associated with astronomical cycles that influence the biosphere. This could be considered a manifestation of physical reductionism, as biological periodicity, if it exists, would be caused by astronomical factors, which obey fundamental physical laws.

To consider this possibility, it is important to take into account the statistical errors in the definition of periodic cycles and the accuracy of the astronomical–biological correlations. For example, in the case of Phanerozoic extinctions, errors of a few Myr are common for defining extinction cycles (Melott & Bambach, 2014). Even if these errors could be considered small in comparison with the duration of an extinction cycle, they are of a magnitude that largely surpasses the ecological timescale and are therefore difficult to explain in terms of biological responses to astronomically triggered environmental changes. The same occurs with chronological correlations between astronomical and extinction cycles. At this scale, methodological constraints in the definition of periodicity are still significant hurdles for making a conclusive assessment.

… life once more comes out as ‘noise’ that does not adhere to fundamental physical laws.

At the Milankovitch scale, however, the statistical errors are significantly smaller and fall within the magnitude of ecological–environmental interactions. But even if biological cycles agree with astronomical periodicities, at least in the case of simple organisms with rapid responses to climate changes, it does not seem to work for more complex and longer lived organisms: the astronomical periodicity is largely distorted by the idiosyncratic responses of different taxa to climatic shifts. This suggests that the possibility of using physics‐like laws could be a matter of scale which progressively decreases when the time band approaches ecological timescales.

## Predictive power

In summary, it can be inferred that inductive prediction power is at a maximum for long‐term biological processes that can be linked to periodic astronomical causes, although the errors associated with the statistical fitting between physical events and biological responses must be taken into account. Inductive prediction power decreases however with the increasing complexity of biological responses that significantly distort astronomical periodicity. This is equivalent to saying that inductive prediction power is directly proportional to the possibility of reducing biological processes to fundamental physical forces. Therefore, life once more comes out as ‘noise’ that does not adhere to fundamental physical laws. This noise is characteristic of long‐term ecological and evolutionary processes, which are inherent to life and require accommodating inductive approaches – hypothesis testing and further generalization – rather than inductive predictions based on fundamental laws. Thus, even in the case of biological processes triggered by well‐known physical causes, the characteristic biological contingency and unpredictability hamper the use of purely reductionist approaches. In addition, humankind – which is also a consequence of evolution on Earth – is a further distortion factor, as it can significantly disrupt future extinction and ecological patterns thus magnifying unpredictability.

### Disclosure statement and competing interests

The author declares that he has no conflict of interest.

## Supporting information


