# Supplementary material for: Inductive prediction in biology: Are long‐term ecological and evolutionary processes predictable?
Source: EMBO Rep. 2022 Jun 20;23(7):e54846. doi: 10.15252/embr.202254846 (PMC9253783; doi:10.15252/embr.202254846)
Supplement: Supplementary file 1 [file EMBR-23-e54846-s001.pdf]

EMBO reports

Inductive prediction in biology

Valentí Rull
DOI: 10.15252/embr.202254846

Corresponding author(s): Valentí Rull (vrull@csic.es)

Review Timeline:

Submission Date:	12th Feb 22
Accepted:	2nd Jun 22

Editor: Holger Breithaupt

Transaction Report:

This manuscript was editorially reviewed with expert advice.

(Note: With the exception of the correction of typographical or spelling errors that could be a source of ambiguity, letters and reports are not edited. Depending on transfer agreements, referee reports obtained elsewhere may or may not be included in this compilation. Referee reports are anonymous unless the Referee chooses to sign their reports.)
